# Why Mayo Clinic Is Embracing the Cloud and What This Means for Clinicians and Researchers

**DOI:** 10.1016/j.mayocpiqo.2021.08.010

**Published:** 2021-09-29

**Authors:** Alexander J. Ryu, Dale R. Magnuson, Thomas C. Kingsley

**Affiliations:** a Division of Hospital Internal Medicine, Department of Medicine, Mayo Clinic, Rochester, MN; b Department of Information Technology, Mayo Clinic, Rochester, MN

### A Brief History of On-Premise Systems and the Rise of Cloud Computing

In our experience, many physicians and researchers may have limited familiarity with the definition and significance of cloud infrastructure, leading to excess concern and confusion. Misguided reservations can then diminish the benefits that a transition to cloud infrastructure could provide for researchers and clinicians. Herein, we seek to illustrate how and why cloud infrastructure can benefit clinical and research workflows. We examine Mayo Clinic’s transition as a case study that could apply to other large academic medical centers.

**Figure 1:**
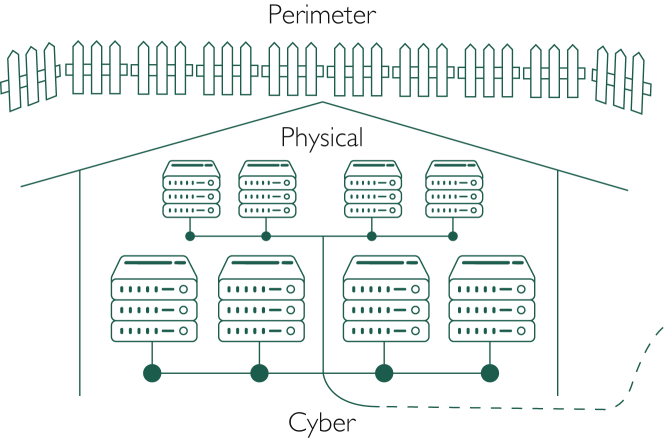
Illustration of perimeter, physical, and cyber security for on-premise servers. Created using free server and fence icons from ClipArtMax^2^ and TheNounProject,^3^ respectively.

Historically, large enterprises, including Mayo Clinic, have handled their computing processes and data storage on-premise, where large warehouses of servers needed to be securely housed and maintained.^1^ Responsibility for the perimeter, physical, and cyber security of these servers (Figure 1) fell to the enterprise owning the servers, and in many cases, this was outside the scope of their primary business. Associated maintenance concerns included ensuring constant power and cooling for the servers, and planning real estate needs accordingly, all of which are fairly removed from the core business of patient care. Researchers who maintain large databases on their own machines, and their collaborators, will be familiar with these challenges, albeit on a relatively smaller scale. For clinicians, this meant that, before EPIC, logging into most clinical applications involved using software that was stored locally (ie, within Mayo Clinic), on their employer’s servers. Data, ie, clicks and keystrokes, exchanged between the clinician, the patient, and the application traveled mainly through a combination of cables and local Internet connections to servers and back again.^1^ As applications became more complex and data generation compounded, this implied the need for Mayo Clinic to purchase, house, and maintain more servers. These purchasing decisions generally involved making some estimate of future needs and purchasing storage and computing resources in excess of current needs. Accordingly, cybersecurity and maintenance challenges multiplied.^4^

**Figure 2:**
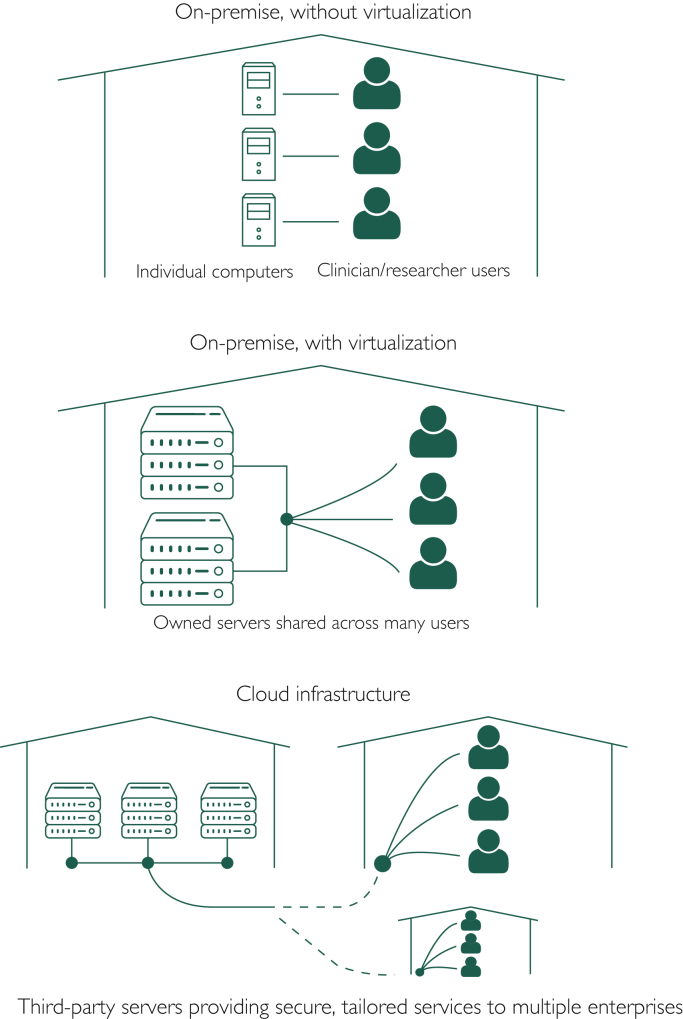
Illustrations of on-premise servers with and without virtualization and cloud infrastructure. Created using free server, desktop, and person icons from ClipArtMax,^2^ Icons8,^6^ and Wikimedia Commons,^7^ respectively.

In the early 2000s, improvements in Internet speeds and virtualization software gave rise to the broad adoption of cloud computing.^5^ Virtualization, which involves the extension of private networks (ie, internal company networks) across public networks (such as the Internet), enabled enterprises to privately and securely run their own networks through a third party’s infrastructure. This availability of third-party hardware and software infrastructure for private use defines “the cloud,” illustrated in Figure 2. For example, Mayo Clinic staff will recall as early as the mid-2000s being able to connect through Mayo’s virtual private network to a virtual machine. This, in essence, was a desktop, separate from the one at their desks, which existed only as a virtual partition of Mayo’s computing resources, in which a single server could provide “unique” desktops to many users. The use of virtualization over time has evolved not only to allow remote access to a health care enterprise’s secure network but also to the outsourcing of server and computing capacity and the associated maintenance. Furthermore, advances in virtualization have allowed for the dynamic allocation of storage and computing power. Third parties such as Amazon and Google, which own and maintain cloud infrastructure, can charge customers based on their real-time needs, allowing customers to pay for storage and computing infrastructure the way they pay for electricity, not rent.^1^ Those who have run websites might recall the decrease in maintenance costs associated with adoption of Amazon Web Services Elastic Compute Cloud for hosting, in which customers (ie, website owners) were charged based on only the traffic to and complexity of their website, as opposed to choosing one of several fixed-cost hosting plans, knowing that if traffic exceeded purchased capabilities, the website would crash.^8^

**Figure 3:**
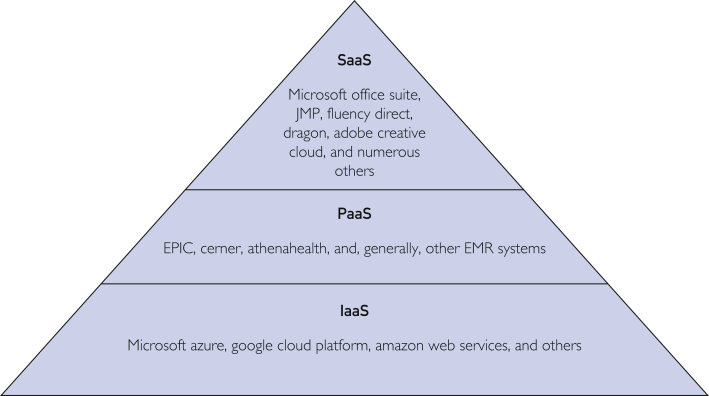
Categories of services provided through cloud computing: infrastructure as a service (IaaS), platform as a service (PaaS), and software as a service (SaaS). EMR indicates electronic medical record.

Although Amazon Web Services, launched in 2006, is generally credited with enabling broad enterprise adoption of cloud computing, competitors were not far behind with launching related products.^9^ Microsoft, Google, Oracle, and IBM have since launched their own related products, each with slightly different strengths and industry focuses.^1^ Furthermore, the range of services provided through cloud computing has expanded significantly, allowing for classification into 3 general categories: infrastructure as a service, which focuses on virtualization and providing the architecture for data storage, transfer, and processing; platform as a service, which provides an operating environment in which customers can develop and run software; and software as a service, which offers customers specific applications to use (Figure 3).^9^ As cybersecurity and system reliability have continued to improve, health systems across the country are beginning to investigate what financial and operational benefits they might stand to gain from cloud adoption.^10^

It is worth noting that although cloud vendors generally promote themselves as using cutting-edge cybersecurity methods, including network monitoring, data encryption, and storage partitioning,^11^^,^^12^ cybersecurity incentives differ somewhat for cloud vendors compared with health care institutions running on-premise systems. In the case of on-premise systems, health care systems are the primary entity with “skin in the game” with respect to securing their patients’ data. With cloud vendors, there could be less incentive to exceed regulatory standards, such as the Health Insurance Portability and Accountability Act, the Health Information Technology for Economic and Clinical Health Act, HITRUST, International Organization for Standardization regulations, and others, relative to an on-premise system; however, cloud vendors’ technical prowess available for engineering robust systems likely exceeds that of most health care systems. Cloud vendors, of course, have significant reputational and legally mandated financial “skin in the game” as well.^13^ Data sharing between cloud vendors and health care systems is further standardized by the use of mandatory Business Associate Agreements, overseen by the Department of Health and Human Services.^13^

### What Cloud Computing Offers to Researchers and Clinicians

Broadly, the transition from on-premise to cloud infrastructure should enable easier access to data and computing power within and even between health care enterprises, which can power the development and deployment of novel analytical tools for research and patient care. In particular, advanced artificial intelligence (AI) techniques, such as deep learning, often require more computing power than what is available at a typical mass-purchased desktop.^1^^,^^10^ Compared with legacy on-premise systems, most cloud providers also offer services in which data can be stored, manipulated, and analyzed in the same location. The novelty of this contrasts with the current standard of requesting a downloaded copy of data and subsequently analyzing it in a location separate from the original, live data table. In addition, the analytical tools available for operating in the cloud have benefitted from the implementation of cutting-edge AI technologies designed for those without computer science backgrounds. Examples of such technologies include optical character recognition, natural language processing, and image analysis.

For clinicians and researchers, this significantly expands the possibilities for developing analytical tools that can be deployed at the bedside. In one example, a Mayo Clinic team is working to automate the IBIS breast cancer risk scoring tool, which has historically been cumbersome to compute due to its need for numerous inputs scattered across a patient’s medical record. In this project, Google’s AI tools are being applied to automatically extract the needed data from a patient’s record and provide the risk score in real time. In the absence of a cloud platform, the best alternative AI tools would likely be less advanced, and the infrastructure needed to find, analyze, and report the data would be prohibitively slow for deployment at the bedside.

In the coming years, Mayo Clinic also plans to migrate and anonymize data from more than 10 million patients from its current enterprise-wide data repository, the Universal Data Platform, to the Google Cloud Platform (GCP). This should enable easier access to data by those interested in training machine learning models and meaningfully shorten the time needed to go from hypothesis, to analysis, to deployment of clinical tools using AI. Clinicians and researchers can presently upload their own data sets containing personal health information as well.

### Accessible AI, Now

As cloud infrastructure has reduced the data storage and computing barriers for clinically applied AI, multiple institutions are working to facilitate access to these tools.^14^^,^^15^ As of September 2020, Mayo Clinic has operationalized its “AI factory,” an initiative that gives any clinician or researcher access to Google’s advanced AI tools with Health Insurance Portability and Accountability Act and HITRUST controls enabled. Mayo Clinic’s AI factory provides staff with an enterprise account in which data sets can be securely uploaded and analyzed using all of Google’s AI tools, with the ultimate aim of developing algorithms that can be deployed in clinical settings. The researcher or clinician must bring funding to pay for the use of Google’s tools, which are billed based on the amount of storage and computing power used. In its present implementation, users must bring their own data sets for upload into their Google account; in subsequent implementations, data will be accessible from within GCP.

Because of the a la carte, elastic nature of GCP’s pricing, budgeting challenges have arisen. For example, although pricing of storage is fairly straightforward (proportional to the size of the data set), estimating a full project budget also requires projections of how long storage and computing resources will be used and which computing resources will be needed. The GCP offers central processing unit–based, graphics processing unit–based, and tensor processing unit–based computing resources. The optimal computing resources will differ from project to project. Although a tensor processing unit is the most powerful and fastest processing unit, it is also the most expensive, and code must be refactored to take full advantage of any efficiencies.

Beyond the selection of processing units, another key consideration affecting project cost is the use of on-demand vs preemptive computing. With on-demand computing, users can leverage Google’s computing resources in real time, with infinite uptime, whereas with preemptive computing, users can be temporarily terminated at any time, specifically, when Google experiences demand peaks. Cost savings can be substantial using preemptive computing, but using this feature requires modifications to a user’s code to prevent significant disruptions if a termination occurs.

Billing for storage and computing is invoiced monthly; the consumption of computing resources must be explicitly switched on and off by the user. To offer some tangible examples, a “small” project for 1 user working 50 hours per week with 1 terabyte (TB) of data stored for 1 year might cost $2000 to $4000 depending on the computing resources used. A larger project for 6 users, again working 50 hours per week with 20 TB of data stored over 1 year might cost $25,000 to $55,000, again depending on the computing resources needed. More precise estimates should become available as more projects are completed.

In summary, Mayo Clinic, similar to many large health care systems, is transitioning to cloud infrastructure that is enabling the rapid transfer of data and access to computing power necessary for the deployment of AI tools in clinical practice. From an IT management perspective, using a cloud platform offers access to computing power, cybersecurity, and system maintenance at a lower cost than on-premise alternatives. To be fair, at this time, we are not aware of any publicly available cost savings reports from hospital systems, and the challenge of providing comparable pre- and post-cloud cost estimates would be considerable due to the complexity of accounting for all pre-cloud costs (numerous first- and third-party goods and services) compared with the relative ease of tallying post-cloud costs (a combined bill from the cloud service provider). It is also possible that over time, although the cost of routine functions can decrease, new expenses from novel services can replace them. The benefits of cloud infrastructure are already accessible to researchers and clinicians at places such as Mayo Clinic. As more, institutions and researchers have access to the benefits of cloud-based systems in the coming years, we expect to observe a proliferation of AI and other advanced analytic tools in medical literature and clinical practice.
